# Construction of an interpretable prediction model for poor functional outcome in conservatively managed basal ganglia hemorrhage based on CT radiomics and multiple machine learning algorithms

**DOI:** 10.3389/fneur.2026.1917496

**Published:** 2026-09-04

**Authors:** Can Luo, JieYao Xia, RuQi Qing, ZhaoPeng Zeng, Xiong Deng

**Affiliations:** 1 Department of Neurosurgery, The First Affiliated Hospital of Shaoyang University, Shaoyang, Hunan, China; 2 Department of Neurosurgery, The Traditional Chinese Medicine Hospital of Longhui County, Shaoyang, Hunan, China

**Keywords:** basal ganglia hemorrhage, machine learning, poor functional outcome, radiomics, restricted cubic spline, SHAP

## Abstract

**Objective:**

Among patients with conservatively managed basal ganglia intracerebral hemorrhage (BG-ICH), a 3-month mRS ≥ 4 signals a poor functional outcome. Routine non-contrast CT reveals intralesional density heterogeneity, yet conventional clinical indicators largely overlook this information, weakening long-term prognostic accuracy.

**Methods:**

We retrospectively enrolled 254 conservatively treated BG-ICH patients and randomly split them into training and internal validation sets (7:3). Clinical variables were compared between outcome groups, and radiomic features were extracted from admission plain CT. Univariate filtering plus LASSO regression isolated robust predictors. Ten machine learning classifiers, including a SuperLearner ensemble, were trained and compared head-to-head. The best random forest (RF) model was assessed via ROC, calibration curves, decision curve analysis, and the KS test. SHAP analyses unpacked feature contributions and nonlinear links to continuous mRS scores.

**Results:**

After dimension reduction, 14 predictive variables—mostly wavelet radiomic features—were retained. RF delivered the highest AUC (0.809), rising to a bootstrap-corrected 0.867 (95% CI, 0.797–0.929), together with good calibration and net clinical benefit. Admission GCS score and key wavelet markers dominated prognostic importance.

**Conclusion:**

This interpretable CT radiomics-based random forest model can stably predict poor functional outcomes at 3 months in patients with basal ganglia intracerebral hemorrhage receiving conservative treatment. Following adequate external cohort validation, this model may assist in individualized risk stratification and serve as a reference for subsequent clinical evaluations. At present, the model cannot be directly implemented in clinical practice, and external validation using independent multicenter cohorts is still required.

## 1 Introduction

Spontaneous basal ganglia intracerebral hemorrhage (BG-ICH) accounts for approximately half of all non-traumatic intracerebral hemorrhage and imposes a heavy socioeconomic burden worldwide due to high rates of long-term severe disability and mortality (1). Most patients with mild-to-moderate hematoma volume receive standardized conservative medical management rather than surgical evacuation. Nevertheless, a substantial proportion of conservatively treated BG-ICH patients eventually develop poor functional outcome, defined as a modified Rankin Scale (mRS) score ≥4 (severe disability or death) at follow-up (2). Early identification of patients at high risk of unfavorable prognosis is critical for timely intensified blood pressure control, neurological monitoring and early rehabilitation to improve long-term functional recovery (3).

Traditional clinical risk markers including baseline hematoma volume, coagulation indicators and the ICH score cannot capture subtle intralesional density heterogeneity invisible on conventional CT scans, which limits their predictive accuracy for long-term functional prognosis (4, 5). Radiomics converts pixel-level CT images into high-dimensional quantitative morphological, texture and wavelet-transformed metrics, enabling non-invasive quantification of microstructural heterogeneity within hematoma lesions that corresponds to underlying pathological changes such as uneven blood clotting and microvascular leakage (6). Over the past decade, radiomics combined with machine learning has been widely applied to build individualized cerebrovascular predictive models, overcoming statistical limitations of conventional single-variable regression for high-dimensional imaging datasets (7).

However, most existing ICH radiomics studies only adopt one or two machine learning algorithms without head-to-head horizontal comparison across multiple classifiers, and few investigations integrate the SuperLearner weighted ensemble framework to optimize predictive performance (8). Additionally, the “black-box” nature of most machine learning models severely hinders clinical translation, as clinicians cannot intuitively interpret the logic behind model risk judgments (9). SHapley Additive exPlanations (SHAP), a game theory-based interpretable artificial intelligence method, simultaneously quantifies global feature importance and individual-level predictive contributions, resolving the interpretability bottleneck of complex tree-based machine learning models (10, 11).

To date, systematic research integrating CT radiomics, multi-algorithm screening, SuperLearner ensemble modeling, and multi-dimensional SHAP plus exploratory DAG-based pathway analysis specifically for poor functional outcome prediction in conservatively managed BG-ICH patients remains scarce. Accordingly, this retrospective cohort study enrolled consecutive patients receiving pure conservative treatment after BG-ICH onset. After radiomic feature extraction and multi-step dimensionality reduction, 10 mainstream machine learning models were constructed and comprehensively evaluated from discrimination, calibration and clinical net benefit perspectives. The optimal RF model was further interpreted via SHAP, counterfactual analysis and exploratory DAG-based pathway analysis to identify core predictive biomarkers and their dose-effect relationships with poor prognosis. This study aims to develop an interpretable non-invasive predictive tool to assist clinicians in early risk stratification and individualized intervention for conservatively treated BG-ICH patients.

## 2 Methods

### 2.1 Study population and outcome definition

This was a single-center retrospective observational study that collected and analyzed medical records of patients diagnosed with spontaneous basal ganglia hemorrhage who were treated at our center between January 2021 and January 2026.

Inclusion criteria: (1) spontaneous basal ganglia hemorrhage confirmed by plain CT within 24 h after symptom onset; (2) received standardized conservative therapy without craniotomy or minimally invasive hematoma drainage; (3) complete baseline CT images, clinical data and three-month follow-up records; (4) bleeding not caused by trauma, intracranial tumor or vascular malformation. Exclusion criteria: (1) severe CT artifacts interfering with accurate hematoma delineation; (2) combined hematological diseases and intractable coagulation disorders; (3) lost to follow-up before the three-month endpoint evaluation; (4) received surgical hematoma evacuation during hospitalization.

**Figure 1:**
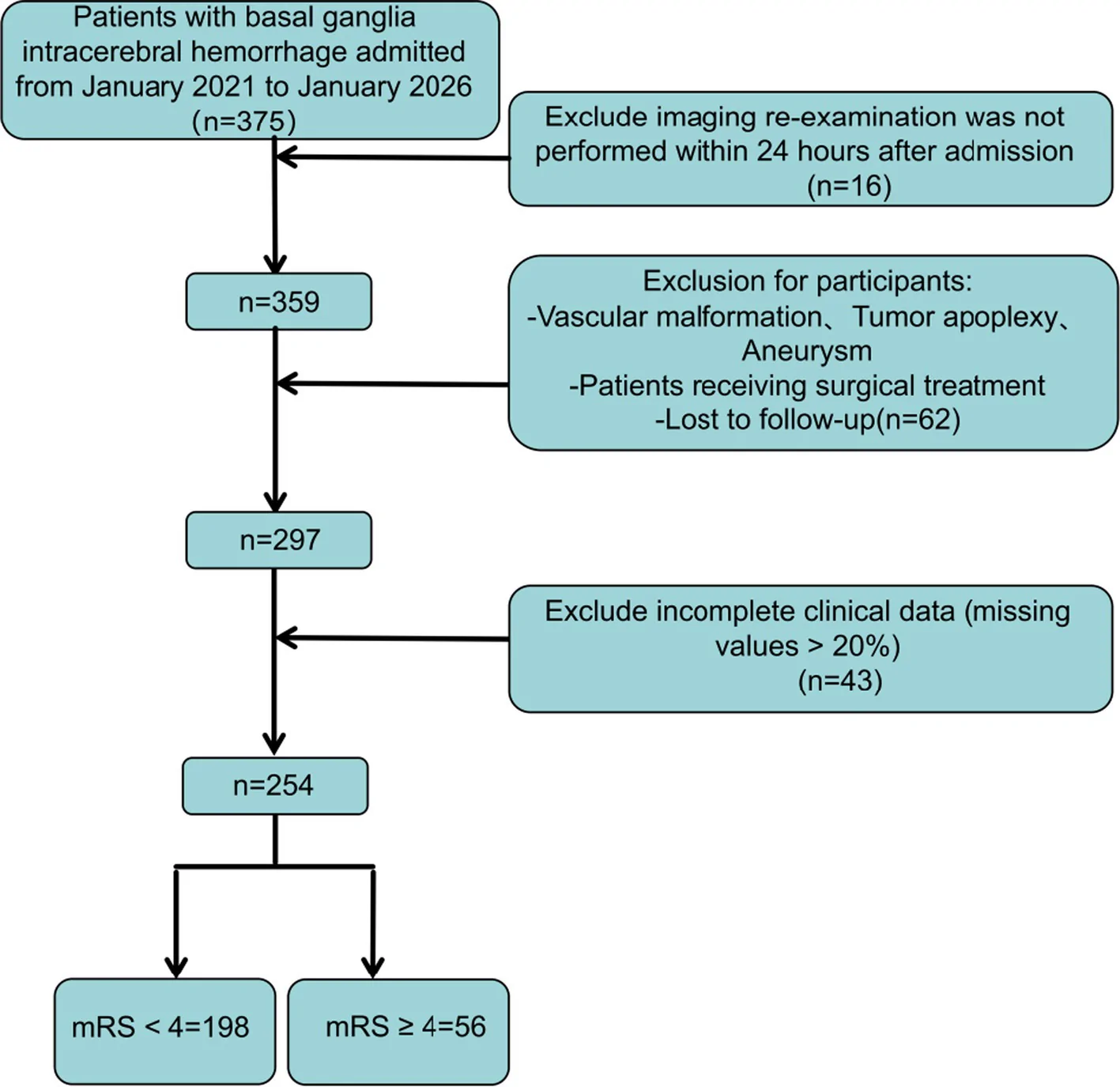
Patient flow chart.

All participants were followed up via outpatient review or telephone interview 3 months after hemorrhage onset. Poor functional outcome was set as the primary endpoint, defined as permanent inability to complete daily self-care or all-cause death (mRS score ≥ 4). The primary endpoint of this study was poor functional outcome at 3 months after onset, which was operationally defined as a modified Rankin Scale (mRS) score ≥ 4. This cutoff value was predetermined at the study design stage rather than derived *post hoc* from the observed outcome distribution data. An mRS score of 4 indicates moderately severe disability: patients cannot walk independently and require assistance with daily living activities and personal self-care. In contrast, an mRS score of 3 corresponds to moderate disability with retained independent ambulation. This differentiation bears direct clinical implications for determining care intensity and formulating rehabilitation plans. Patients with mRS ≥ 4 generally require long-term care from family caregivers or admission to professional nursing facilities. Subjects were divided into training group and internal validation group through stratified random sampling to balance event rates between subgroups. The study obtained ethical approval. Due to retrospective analysis of de-identified medical data, the ethics committee waived the requirement for individual informed consent. Baseline clinical characteristics were collected and analyzed, including demographic data, vital signs, laboratory indicators, medical history and treatment-related variables. Samples with variable missing rates exceeding 20% were excluded, and multiple imputation was adopted to supplement data for variables with missing values. Measurement data were expressed as mean ± standard deviation, and intergroup differences were compared by independent sample *t*-test. Count data were expressed as number of cases (percentage), and intergroup differences were tested by chi-square test. Two-tailed *p* < 0.05 was defined as statistically significant (Figure 1).

### 2.2 Radiomic feature extraction and preprocessing

Two attending neurosurgeons manually outlined hematoma regions slice by slice on thin-slice baseline CT scans. The delineation scope was strictly limited to hemorrhagic parenchyma to avoid peripheral edema and normal brain tissue. Any inconsistent ROI demarcation was resolved by a senior chief physician with decades of clinical experience to guarantee unified drawing standards. CT images were resampled to isotropic 1 × 1 × 1 mm^3^ voxels using B-spline interpolation, discretized with a bin width of 25 HU, and normalized via *Z*-score within the PyRadiomics framework. The open-source PyRadiomics software was applied to extract radiomic indicators, including first-order statistics, morphological parameters, texture features, and multi-scale wavelet transformed metrics. After extraction, all continuous variables — both radiomic features and clinical predictors — were standardized to zero mean and unit variance. Scaling parameters were derived exclusively from the training set and then applied to the validation set, preventing data leakage; categorical variables were not scaled. Simultaneously, clinical variables within the first 24 h of admission were collected: age, gender, history of hypertension and diabetes, smoking and drinking status, admission GCS score, admission NIHSS score, intraventricular hemorrhage, body temperature, body mass index, heart rate, respiratory rate, baseline hematoma volume, and time from symptom onset to CT scan. Univariate statistical tests were performed to screen differential features according to data distribution types. Variables meeting the screening threshold were retained for subsequent LASSO dimensionality reduction.

### 2.3 LASSO regression for feature selection

LASSO regression combined with five-fold cross-validation was used to remove highly correlated redundant features and reduce model overfitting risk. Cross validation error curves were drawn to screen the optimal penalty coefficient. Variables with non-zero regression coefficients under the optimal λ value were selected as final predictive indicators for model construction.

### 2.4 Construction of machine learning models

Nine independent machine learning classifiers were trained on the training dataset: Logistic Regression, AdaBoost, Random Forest, XGBoost, Support Vector Machine, K-Nearest Neighbor, Gradient Boosting Machine, Decision Tree and Neural Network. Given the modest sample size, all classifiers were trained with standard default configurations to ensure fair comparison and avoid tuning-driven overfitting. Random Forest used 500 trees; XGBoost used 100 rounds (max_depth = 6, eta = 0.3); SVM used RBF kernel (*C* = 1); KNN used *k* = 5; GBM used 500 trees (interaction.depth = 3); AdaBoost used 100 iterations; Decision Tree and Logistic Regression used defaults; Neural Network used a single 5-node hidden layer (decay = 0.01, maxit = 1,000). On this basis, the SuperLearner integrated model was established by weighted combination of the nine base learners, and bar charts were used to display the weight distribution of each base algorithm.

### 2.5 Model performance assessment

Model performance was evaluated from four dimensions: discrimination, comprehensive predictive efficiency, calibration and clinical practicability. Discriminative ability: ROC curves and corresponding AUC values were plotted and calculated. DeLong test was used for pairwise comparison of all algorithms and visualized by heatmap. Bootstrap resampling was carried out on the optimal random forest model to obtain corrected discriminative index and confidence interval. Comprehensive efficiency: Radar charts were drawn to compare AUC, accuracy, F1-score, recall and PPV of all models, with independent bootstrap confidence intervals attached to each metric. Calibration performance: Calibration curve of random forest was drawn to compare predicted risk and actual poor outcome incidence. Clinical utility: Decision curve analysis quantified net clinical benefit under different risk thresholds compared with treat-all and treat-none reference strategies. KS test was used to screen optimal risk cutoff and evaluate the model’s ability to distinguish high and low-risk patients.

### 2.6 Regression models for continuous mRS outcome

Eight prediction algorithms covering traditional linear regression and machine learning approaches were developed to predict continuous clinical endpoint, including Linear Regression, Elastic Net, single Decision Tree, K-nearest neighbor (KNN), Support Vector Machine (SVM), Random Forest (RF), Gradient Boosting Machine (GBM), and XGBoost. All enrolled samples were randomly split into training cohort (70%) and independent test cohort (30%) at a fixed random seed. For each algorithm, three types of diagnostic plots were generated to evaluate model fitting, Histogram of predicted values: Blue bars denoted predicted value distribution of training set, gray bars represented test set, to compare the distribution consistency between two subsets; Observed vs. predicted scatter plot: *X*-axis = model-predicted value, *Y*-axis = actual observed value, the black dashed line represented ideal perfect fitting (predicted value = actual value). Coefficient of determination (R^2^) and overall mean absolute error (MAE) were annotated to quantify fitting efficiency; Residual distribution plot: Residual = actual value − predicted value, horizontal black line at residual = 0 was the reference baseline. Training-set MAE (MAE_train_) and test-set MAE (MAE_test_) were calculated to assess prediction error and overfitting risk; smaller gap between MAE_train_ and MAE_test_ indicated better generalization ability.

### 2.7 SHAP and causal inference analysis

SHAP algorithm was applied on the optimal RF model for multi-layer interpretation, Global interpretation: Feature importance ranking by mean absolute SHAP values and SHAP summary plots. Marginal effect: SHAP dependence plots depicting continuous changes in feature values versus corresponding SHAP scores and critical thresholds. Individual interpretation: Waterfall and force plots for three representative patients to illustrate feature-specific positive/negative risk contributions at single-case level. Exploratory pathway analysis: Directed Acyclic Graph (DAG) mapped hypothesized pathways linking demographics, clinical covariates, radiomic markers and poor functional outcome outcomes; counterfactual SHAP analysis was conducted on borderline patients to verify potential associations of key features. It should be noted that SHAP quantifies feature importance within the trained model and does not infer causality. Interpretations should be viewed as hypothesis-generating rather than confirmatory of biological mechanisms.

### 2.8 TRIPOD reporting compliance

This study was reported in accordance with the Transparent Reporting of a Multivariable Prediction Model for Individual Prognosis or Diagnosis (TRIPOD) statement. As this study employed machine learning methods, the TRIPOD-AI extension was additionally consulted to ensure comprehensive reporting of artificial intelligence-related methodology.

### 2.9 Statistical software

All statistical analyses, feature extraction, machine learning construction and SHAP/RCS computation were completed using Python 3.9 and R 4.2.1. Two-tailed *p* < 0.05 indicated statistical significance. Measurement data were expressed as mean ± standard deviation (x±s) , and independent-samples t test was used for intergroup comparisons. Enumeration data were presented as number of cases (percentage) n(%) , and the $\chi^{2}$ test was adopted for intergroup comparisons. A *P*-value < 0.05 was considered statistically significant.

## 3 Results

### 3.1 Baseline characteristics of enrolled retrospective patients

A total of 254 eligible conservatively treated basal ganglia hemorrhage patients were included for analysis, with 198 patients in the favorable outcome group (mRS ≤ 3) and 56 patients in the poor outcome group (mRS ≥ 4). The baseline clinical characteristics of the two groups are shown in Table 1. There was no significant difference between the two groups in age, gender, heart rate, respiratory rate, hypertension history, anticoagulant/antiplatelet use, number of comorbidities, eGFR and D-Dimer (all *p* > 0.05). Statistically significant differences were observed between the two groups in body temperature, BMI, GCS score, intraventricular hemorrhage, IV antihypertensive use, ALT, AST and admission mRS score (all *p* < 0.05). Patients in the poor outcome group had significantly lower GCS score, higher BMI, higher body temperature, higher incidence of intraventricular hemorrhage, higher ALT and AST levels, and higher admission mRS score compared with the favorable outcome group (Table 1).

**Table 1:** Baseline clinical characteristics of patients with favorable and poor outcome.

Variable	Good outcome (*n* = 198)	Poor outcome (*n* = 56)	Total (*n* = 254)	*p*.value
Gender				0.546
Male	133 (67%)	40 (71%)	173 (68%)	
Female	65 (33%)	16 (29%)	81 (32%)	
Age (years)	64 (13)	62 (13)	64 (13)	0.214
Temperature (°C)	36.73 (0.40)	36.80 (0.39)	36.74 (0.40)	0.038
BMI (kg/m^2^)	23.2 (4.0)	25.6 (6.0)	23.6 (4.5)	0.034
Heart Rate (bpm)	80 (16)	85 (19)	81 (17)	0.138
Respiratory Rate (bpm)	20.1 (3.4)	19.5 (3.5)	20.0 (3.4)	0.208
GCS Score	11.8 (3.2)	8.3 (3.3)	11.0 (3.6)	0.000
Intraventricular Hemorrhage	27 (14%)	26 (46%)	53 (21%)	0.000
Hypertension History	144 (73%)	46 (82%)	190 (75%)	0.152
Anticoagulant/Antiplatelet Use	16 (8.1%)	8 (14%)	24 (9.4%)	0.161
Comorbidities (number)				0.737
0	37 (19%)	8 (14%)	45 (18%)	
1	76 (38%)	23 (41%)	99 (39%)	
2	62 (31%)	17 (30%)	79 (31%)	
3	16 (8.1%)	4 (7.1%)	20 (7.9%)	
4	7 (3.5%)	4 (7.1%)	11 (4.3%)	
IV Antihypertensive	125 (63%)	43 (77%)	168 (66%)	0.057
ALT (U/L)	23 (19)	27 (18)	24 (19)	0.027
AST (U/L)	30 (22)	32 (17)	30 (21)	0.030
eGFR (mL/min/1.73m^2^)	81 (35)	84 (31)	81 (34)	0.399
D-Dimer	0.99 (2.41)	1.47 (3.52)	1.11 (2.73)	0.815
Admission mRS Score	1.55 (1.10)	5.00 (0.85)	2.31 (1.78)	0.000

### 3.2 Feature selection

**Figure 2:**
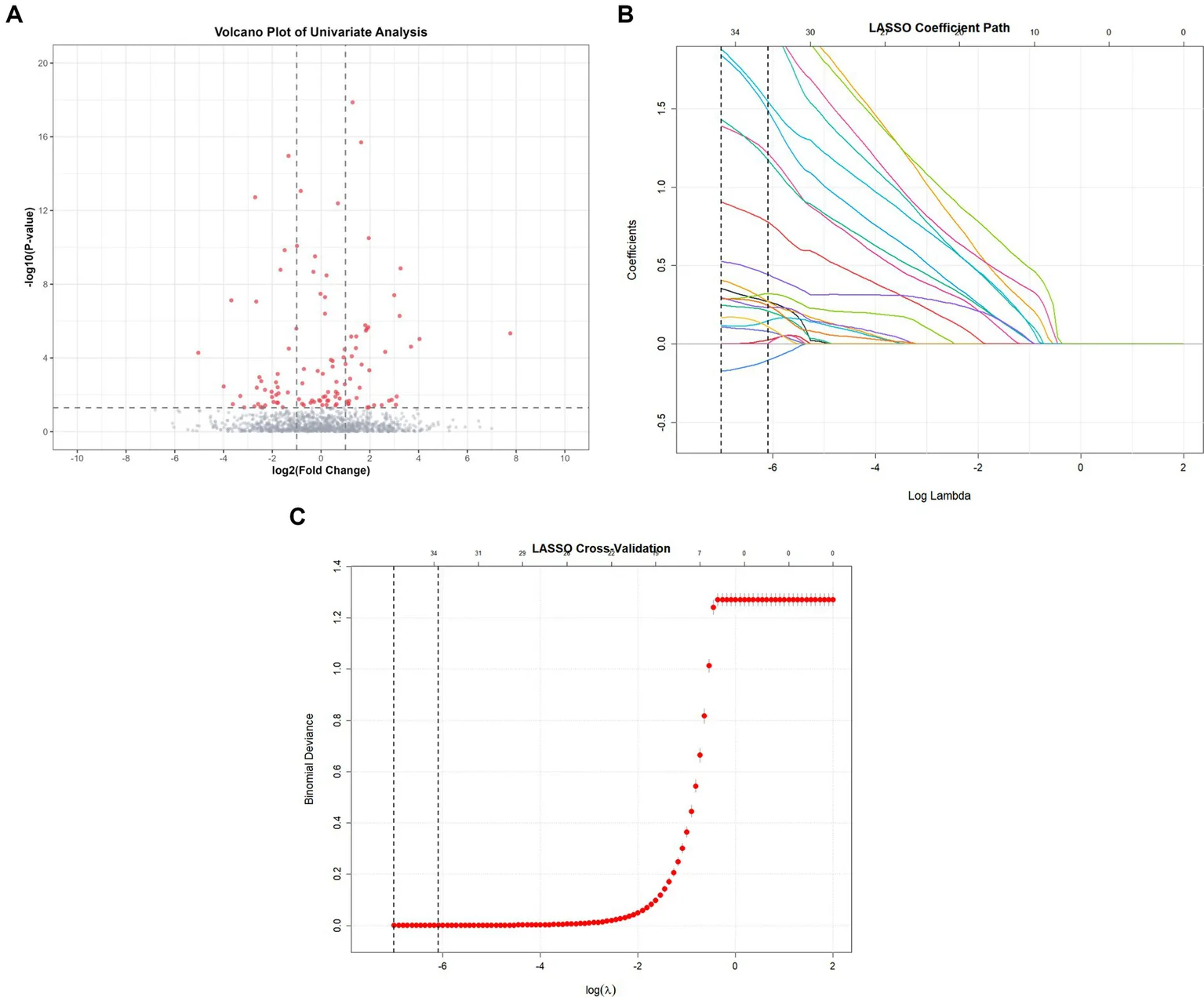
**(A)** Volcano plot displaying univariate statistical screening results of radiomic features between poor functional outcome and non-poor functional outcome groups. **(B)** LASSO coefficient shrinkage curve for radiomic feature screening. **(C)** Cross-validation binomial deviance curve for LASSO regularization parameter screening.

From the initial 1,215 features, univariate screening identified 137 significantly differential radiomic features. After LASSO regularization at the optimal log(λ) ≈ −6, 14 predictors were retained: eight wavelet features, age, body temperature, BMI, heart rate, respiratory rate, and GCS (Figure 2).

### 3.3 Comparative discriminative performance of all models

**Figure 3:**
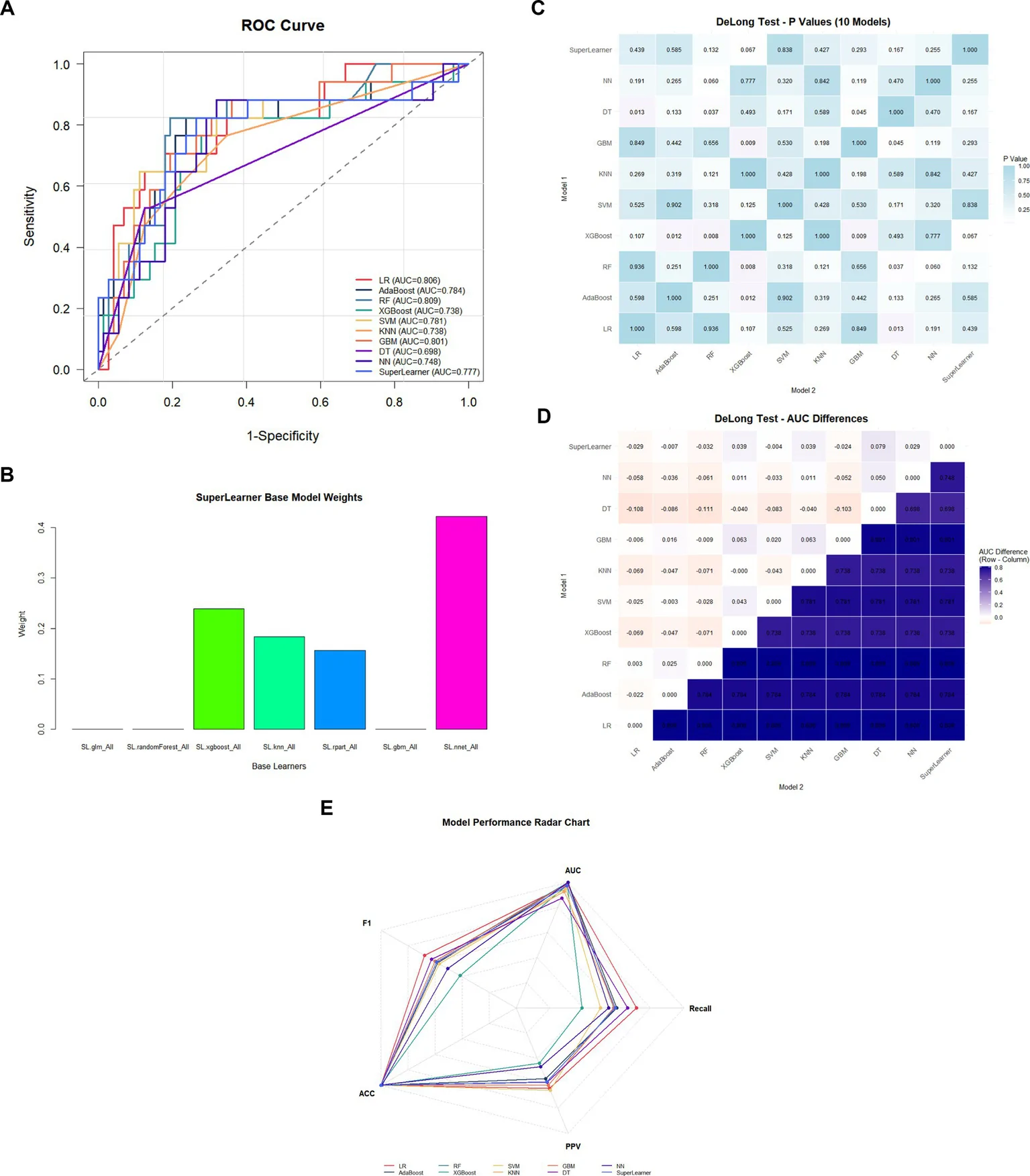
**(A)** Receiver operating characteristic (ROC) curves of 10 machine learning models for predicting acute poor functional outcome after BG-ICH in the training cohort. **(B)** Radar chart summarizing five predictive evaluation metrics across all 10 machine learning models for BG-ICH poor functional outcome prediction. **(C)** Heatmap of *P* values from pairwise DeLong test for AUC comparison among 10 predictive machine learning models. **(D)** Heatmap of pairwise AUC differences (row value minus column value) calculated from DeLong test among 10 machine learning prediction models. **(E)** Weight distribution of base learners in the constructed SuperLearner ensemble model.

Discriminative and comprehensive evaluation indicators with corresponding confidence intervals of all 10 models are summarized in Table 2. Random Forest had the highest AUC value, followed by Logistic Regression and Gradient Boosting Machine. Decision Tree showed the weakest discriminative capacity. Pairwise DeLong test confirmed random forest was significantly better than several other algorithms, while no statistical difference was found among random forest, logistic regression and gradient boosting machine. Logistic Regression and Support Vector Machine achieved the highest accuracy. Logistic Regression also had favorable F1-score and recall. The SuperLearner integrated model failed to produce better discriminative performance than top single tree-based algorithms. Weight analysis of SuperLearner showed neural network and XGBoost occupied major fusion weights, while random forest and logistic regression received negligible weight during model integration (Figure 3 and Table 2).

**Table 2:** Predictive performance of machine learning models for three-month poor functional outcome in conservatively managed basal ganglia hemorrhage.

Model	AUC (95%CI)	Accuracy (95%CI)	F1-score (95%CI)	Recall (95%CI)	PPV (95%CI)
LR	0.806 (0.688–0.908)	0.820 (0.742–0.888)	0.556 (0.345–0.718)	0.588 (0.352–0.818)	0.526 (0.313–0.750)
AdaBoost	0.784 (0.646–0.902)	0.787 (0.697–0.865)	0.457 (0.256–0.647)	0.471 (0.235–0.722)	0.444 (0.227–0.684)
RF	0.809 (0.682–0.914)	0.809 (0.719–0.899)	0.485 (0.261–0.700)	0.471 (0.261–0.722)	0.500 (0.250–0.778)
XGBoost	0.738 (0.583–0.864)	0.753 (0.663–0.843)	0.313 (0.083–0.511)	0.294 (0.077–0.526)	0.333 (0.091–0.571)
SVM	0.781 (0.616–0.915)	0.820 (0.741–0.888)	0.467 (0.222–0.667)	0.412 (0.182–0.667)	0.538 (0.250–0.800)
KNN	0.738 (0.609–0.864)	0.798 (0.708–0.876)	0.471 (0.231–0.667)	0.471 (0.222–0.714)	0.471 (0.227–0.714)
GBM	0.801 (0.682–0.911)	0.809 (0.730–0.888)	0.485 (0.263–0.667)	0.471 (0.231–0.714)	0.500 (0.250–0.750)
DT	0.698 (0.550–0.838)	0.798 (0.708–0.876)	0.500 (0.258–0.686)	0.529 (0.273–0.778)	0.474 (0.231–0.706)
NN	0.748 (0.586–0.879)	0.742 (0.640–0.820)	0.378 (0.158–0.565)	0.412 (0.182–0.688)	0.350 (0.136–0.556)
SuperLearner	0.777 (0.631–0.906)	0.798 (0.708–0.876)	0.471 (0.235–0.667)	0.471 (0.222–0.722)	0.471 (0.222–0.727)

AUC, area under receiver operating characteristic curve; CI, Bootstrap confidence interval; SE, standard error of AUC; ACC, overall accuracy; F1, composite evaluation score; Recall, sensitivity; PPV, positive predictive value.

### 3.4 Bootstrap internal validation of random forest

**Figure 4:**
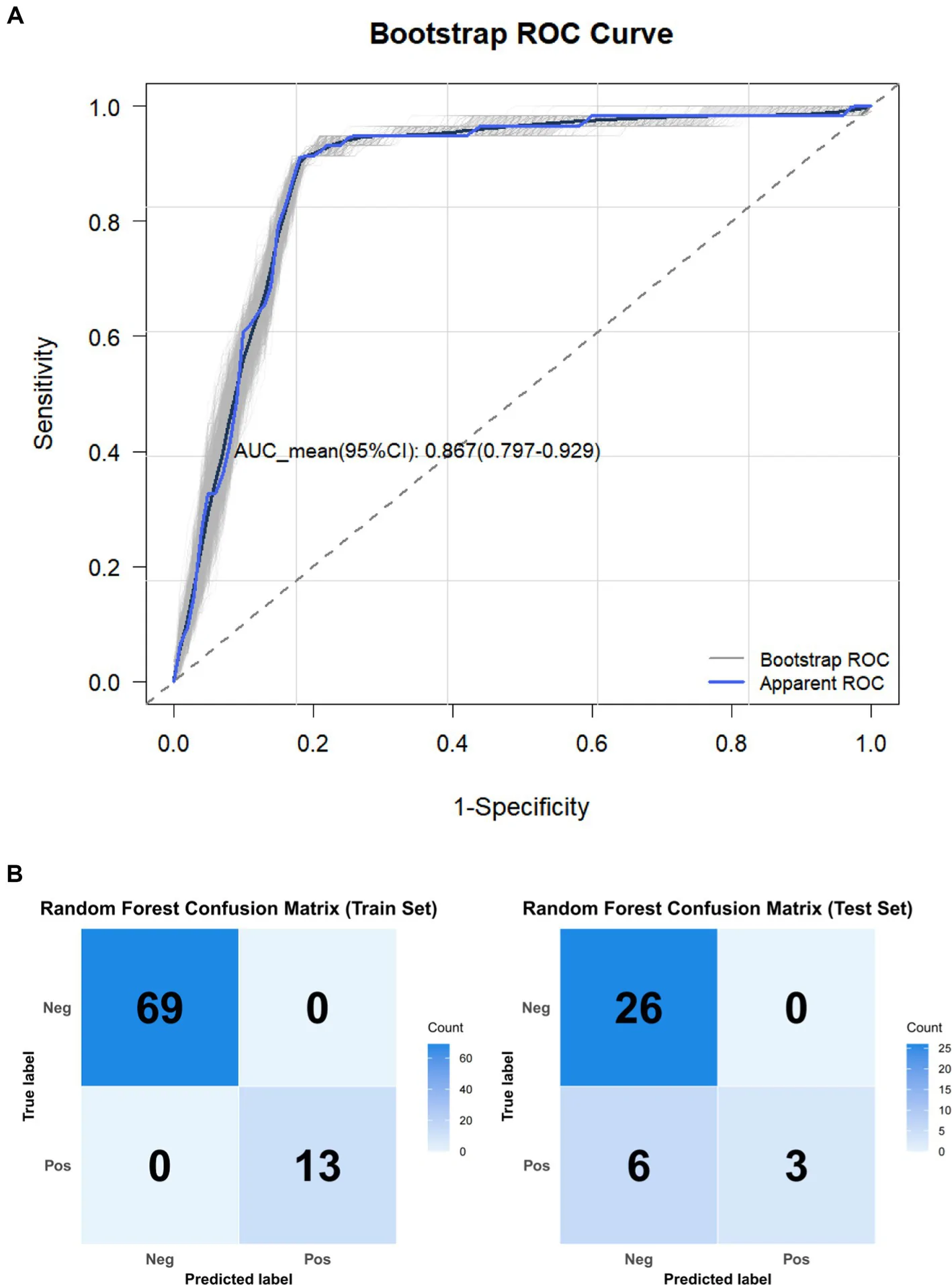
**(A)** Bootstrap-corrected ROC curve of the optimal RF prediction model for BG-ICH poor functional outcome. **(B)** Confusion matrices of the optimal random forest model on training and test cohorts for BG-ICH poor functional outcome prediction.

Thousand times Bootstrap resampling internal validation was conducted on random forest. Corrected discriminative index indicated stable model performance, and resampled AUC distribution approximated normal distribution without obvious overfitting. Confusion matrix of the validation set showed high overall accuracy, good sensitivity and perfect specificity, with only a small number of actual poor outcome patients misclassified as low-risk cases (Figure 4).

### 3.5 Calibration, DCA and KS results

**Figure 5:**
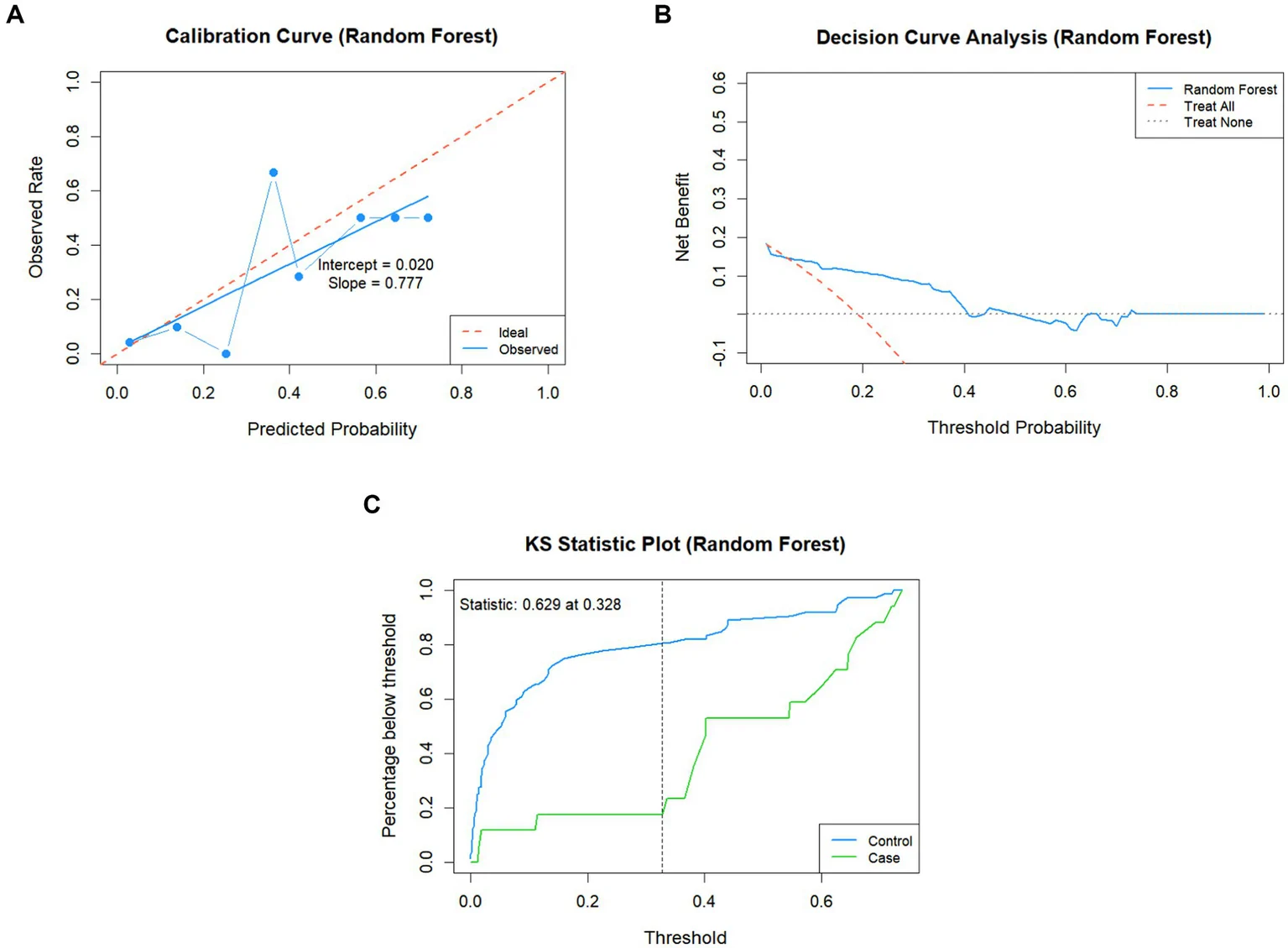
**(A)** Calibration curve of the optimal random forest prediction model for early poor functional outcome after basal ganglia intracerebral hemorrhage. **(B)** Decision curve analysis (DCA) of the optimal random forest model for predicting poor functional outcome after basal ganglia intracerebral hemorrhage. **(C)** KS curve of the optimal random forest model for BG-ICH poor functional outcome prediction.

The random forest calibration curve was highly consistent with the ideal reference line, indicating good matching between predicted risk and actual poor outcome incidence. Decision curve analysis showed the model brought higher net clinical benefit than universal intervention and blank management within common risk ranges. KS test identified an appropriate optimal cutoff value with strong ability to separate patients with favorable and poor prognosis (Figure 5).

### 3.6 Fitting performance of continuous mRS regression models

**Figure 6:**
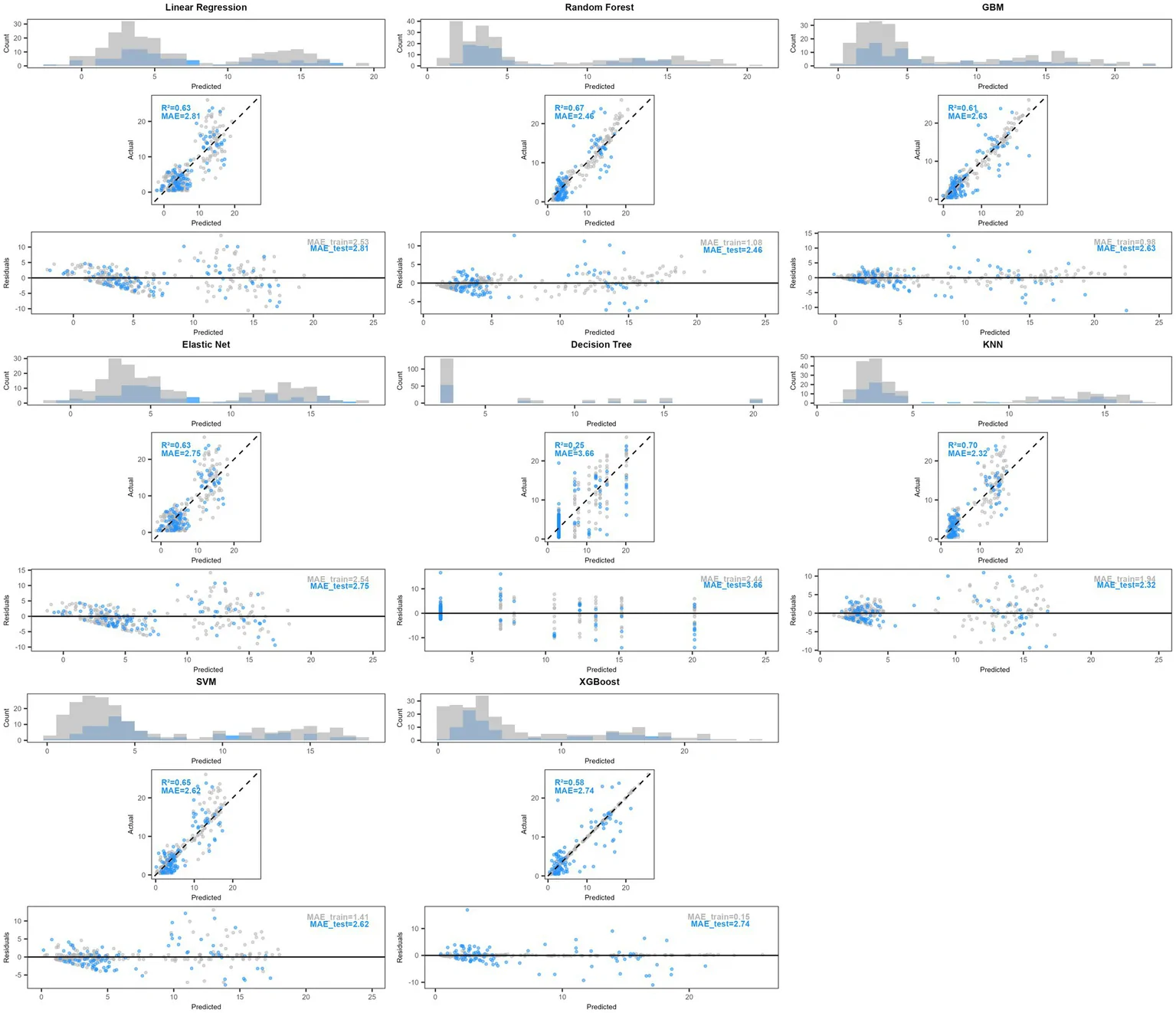
Observed-predicted scatter plots and residual distribution plots of eight regression algorithms for continuous clinical outcome prediction. The upper subgraph shows the frequency distribution of predicted values (blue: Training set; gray: Test set); the middle scatter plot compares predicted value and actual observed value (black dashed line = ideal fitting); the bottom subgraph presents residual distribution against predicted value, with MAE_train_ and MAE_test_ annotated for each model.

Eight regression models were constructed to predict continuous three-month mRS values. Random Forest achieved the best overall fitting effect, with predicted values highly consistent with actual scores and evenly distributed residuals, and a small gap between training and test set error values. Ensemble tree algorithms had moderate fitting effects. Traditional linear models showed limited predictive capacity, while single decision tree performed worst with scattered scatter points and irregular residual distribution (Figure 6).

### 3.7 SHAP results

**Figure 7:**
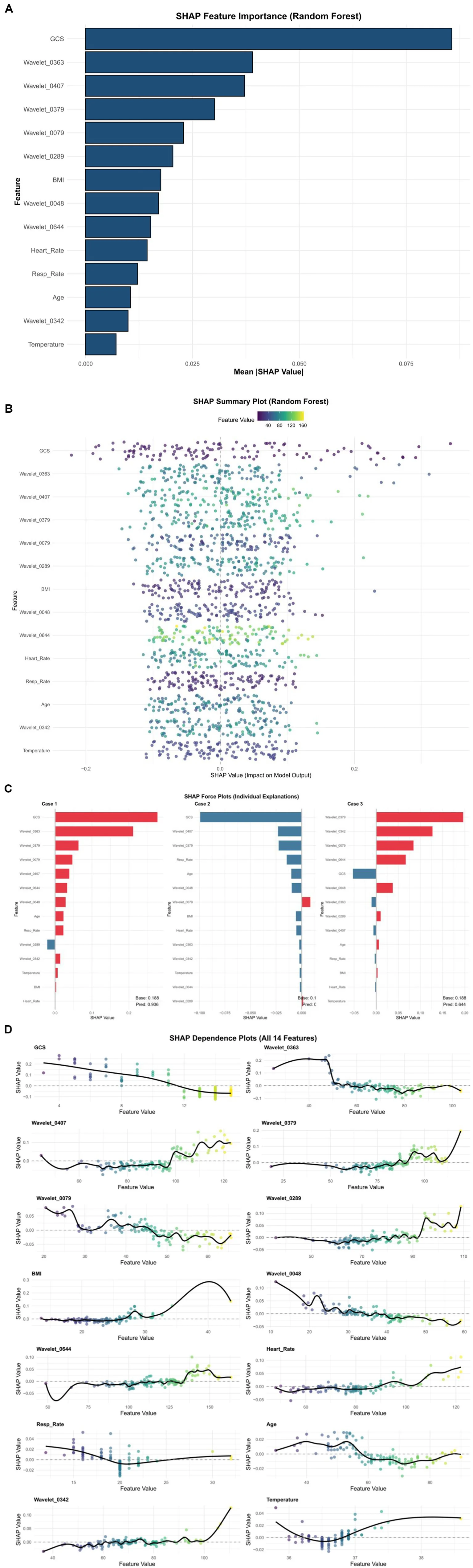
**(A)** SHAP-based feature importance of random forest model. **(B)** SHAP summary plot of random forest model. **(C)** Individual SHAP force plots for three representative patients. **(D)** SHAP dependence plots of all predictive features for poor functional outcome prediction.

Global SHAP importance ranking showed admission GCS score, age, heart rate, respiratory rate and wavelet radiomic features were core prognostic predictors, among which GCS score had the greatest influence. Lower GCS value corresponded to higher risk of poor outcome, while higher GCS value played a protective role. Most wavelet features had obvious threshold effects, shifting from protective to risk-promoting effects once exceeding critical values. SHAP force plots of typical patients showed different dominant risk factors for individuals, but wavelet imaging markers were always the main risk drivers, consistent with the hypothesis that intralesional density heterogeneity, as captured by wavelet radiomic features, may reflect underlying pathological variation such as uneven blood coagulation and microvascular leakage. However, SHAP values reflect associative contributions within the model framework and do not establish direct causal or biological mechanisms. Among clinical indicators shown in the dependence plots, admission GCS carried the highest prognostic weight, with lower GCS associated with higher risk of long-term disability and death; heart rate > 90 beats/min, body temperature > 37 °C, and BMI > 30 all elevated the risk of poor outcome, whereas low respiratory rate and middle-aged patients (40–60 years) had worse prognosis. Each wavelet imaging feature exhibited a distinct threshold: low expression of Wavelet_0363, 0407, 0379, 0289, and 0342 exerted protective effects, with risk increasing once exceeding the threshold; both high and low values of Wavelet_0079 increased risk, with moderate levels associated with the best prognosis; higher values of Wavelet_0048 predicted better prognosis; Wavelet_0644 showed only mildly diminished protective effects (Figure 7).

### 3.8 DAG and counterfactual analysis

**Figure 8:**
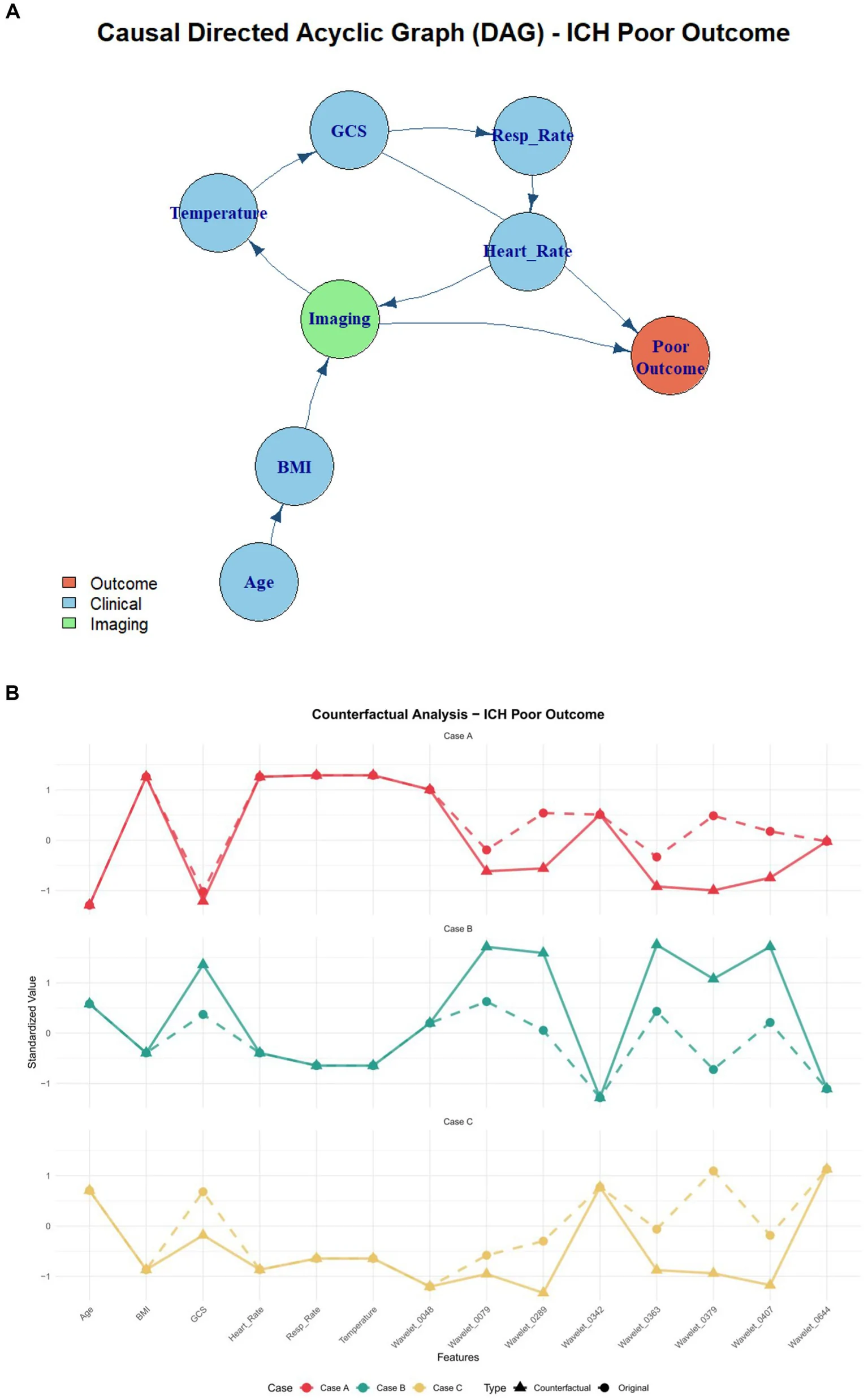
**(A)** Causal directed acyclic graph (DAG) for predictors of intracerebral hemorrhage poor functional outcome risk. **(B)** Counterfactual analysis for a borderline false-positive sample.

The directed acyclic graph was constructed to explore hypothesized pathways connecting demographic, clinical, and radiomic variables to long-term functional outcome, informed by domain knowledge. In this exploratory framework, age and body mass index were positioned as potential upstream factors that may influence hematoma texture features and vital signs; admission GCS score was linked to heart rate and respiratory stability; wavelet radiomic markers and vital signs were modeled as potential direct contributors to three-month prognosis. These pathways are hypothesis-generating and do not establish causal relationships. Counterfactual parallel plot simulation demonstrated that adjusting core imaging markers could greatly change the predicted risk of borderline patients, which supported the hypothesis that intralesional hematoma heterogeneity may carry independent prognostic information (Figure 8).

## 4 Discussion

### 4.1 Principal findings and literature comparison

In this retrospective radiomic research focusing on poor functional outcome after basal ganglia intracerebral hemorrhage, after multi-step feature selection including univariate statistical filtering and LASSO penalized regression, 14 robust radiomic, clinical and hematoma-related quantitative features were finally screened out for model construction, among which wavelet-transform-derived radiomic features dominated the top-ranked predictive contributors verified by subsequent SHAP global importance ranking (12). Multi-index comprehensive evaluation covering ROC-AUC, accuracy, F1-score, recall rate and PPV consistently identified random forest (RF) as the optimal standalone predictive classifier, while SuperLearner ensemble model failed to achieve a statistically superior discriminative ability compared with high-performance single models including RF and logistic regression via DeLong test of AUC differences (13). Internal Bootstrap validation, calibration curve and DCA decision curve analysis further confirmed robust discrimination, favorable calibration and considerable net clinical benefit of the finalized RF model, and subsequent multi-dimensional SHAP visualization successfully realized transparent global and individualized local interpretation for the black-box RF algorithm (14). Consistent with several previous radiomic studies on ICH hematoma expansion prediction, wavelet-decomposed texture features derived from baseline non-enhanced CT occupied dominant predictive weight in our final predictive signature, thereby contributing to poor prognosis (15). Wavelet transformation can decompose original CT hematoma image into multi-frequency sub-images to capture subtle microscopic inhomogeneity of hematoma internal density which corresponds to uneven blood coagulation, ongoing microvascular oozing and heterogeneous fibrin formation inside acute hematoma, and these invisible microstructural variations are closely correlated with the risk of poor functional outcome (16). Different from prior studies that simply adopted one or two machine learning algorithms for modeling, the present work systematically compared 10 mainstream supervised classifiers and adopted SuperLearner weighted ensemble strategy, providing rigorous head-to-head algorithm evidence for future radiomic modeling of BG-ICH complications (17).

### 4.2 Clinical value of interpretable analysis

Traditional radiomic machine learning studies mostly stop at model performance verification without credible model interpretation, restricting clinical conversion from laboratory data to bedside application (18). In the current study, a comprehensive SHAP analytical system including global importance bar chart, SHAP summary dot plot, partial dependence plot, individual waterfall plot and force plot clearly quantified the positive/negative risk contribution of each core feature: elevated values of dominant wavelet features significantly increased the risk of poor functional outcome by shifting the SHAP value from a negative protective effect to a positive risk-promoting effect, whereas baseline hematoma volume, vital signs, BMI, and GCS also demonstrated considerable predictive influence within limited numerical intervals (19). Individual SHAP case visualization directly explained the specific feature-driven reasons behind the high risk of poor functional outcome in representative BG-ICH patients, helping neurosurgeons understand the model’s risk-judgment logic instead of blindly trusting black-box output results (20). Additionally, exploratory directed acyclic graph (DAG) analysis and counterfactual SHAP analysis further mapped hypothesized pathways linking clinical baseline factors, macroscopic hematoma indices, microscopic radiomic characteristics, and poor functional outcome. This analysis generates hypotheses about potential mechanistic links that merit further investigation, although the observational design precludes causal conclusions (21).

### 4.3 Reasons for limited improvement of SuperLearner

Unexpectedly, the SuperLearner integrated model did not obtain a significantly improved AUC compared with the optimal single RF model in DeLong statistical comparison, which was partially attributable to heterogeneous performance discrepancy of base learners and limited sample size of internal validation cohort (22). Weight distribution of base learners indicated neural network and XGBoost occupied dominant fusion weight while high-efficiency RF and LR obtained nearly zero weight during SuperLearner optimization process, which meant the ensemble framework overly depended on moderate-performance base algorithms and diluted predictive advantages of top-ranked single models (9). Such phenomenon has also been reported in several recent radiomic predictive studies of cerebrovascular diseases, reminding researchers that ensemble learning cannot guarantee performance improvement blindly and multi-model horizontal screening remains essential before final model confirmation (23).

### 4.4 Clinical implications

This RF radiomic prediction tool relies solely on routine admission non-contrast CT and standard clinical data, without requiring expensive multimodal imaging or invasive laboratory examinations, and is specifically applicable to patients receiving pure conservative medical management without surgical intervention. If externally validated, this model could assist clinicians in rapidly stratifying risk at admission: high-risk patients identified by the model could be considered for intensified blood pressure regulation, close neurological monitoring, and early standardized rehabilitation aimed at reducing the probability of severe long-term disability. Conversely, low-risk patients could potentially avoid unnecessary prolonged ICU observation, thereby helping to optimize limited medical resources (24, 25). The screened core wavelet radiomic biomarkers also suggest novel quantitative imaging hypotheses for basic research on hematoma lysis and secondary brain injury mechanisms (26).

### 4.5 Outcome definition

The choice of mRS ≥ 4 versus the alternative threshold of mRS ≥ 3 as the definition of poor functional outcome warrants discussion. The mRS ≥ 3 threshold, which encompasses moderate-to-severe disability, is more aligned with the concept of ‘loss of functional independence’ and is commonly used in acute stroke trials. Applying mRS ≥ 3 to our cohort would result in a larger proportion of patients classified as having poor outcome, potentially improving statistical power for model training but also increasing outcome heterogeneity. We selected mRS ≥ 4 as the primary endpoint *a priori* because (1) it represents a clinically unequivocal severe disability state requiring substantial assistance for daily activities, (2) from a clinical decision-making perspective, distinguishing patients likely to progress to severe disability or death has greater implications for treatment escalation. Future studies may explore whether radiomic models perform differently under alternative dichotomization thresholds or using ordinal analysis of the full mRS scale.

### 4.6 Limitations

Several limitations of this study require clarification. First, this is a single-center retrospective cohort focusing solely on conservatively treated BG-ICH patients; the model cannot be generalized to patients receiving surgical hematoma evacuation, and independent multicenter prospective external validation is required to verify cross-center robustness (27). Second, only conventional non-contrast CT images were used for radiomic feature extraction; multimodal imaging markers including CTA spot sign and SWI microbleeds were not incorporated due to incomplete clinical data collection, potentially restricting predictive performance (28). Third, partial clinical confounding factors such as irregular antiplatelet/anticoagulant medication history and dynamic early blood pressure fluctuations were not quantified into the final feature set, possibly introducing residual confounding effects (29). Fourth, hematoma ROIs were manually delineated by clinicians instead of automatic deep learning segmentation algorithms, inevitably introducing minor inter-observer segmentation bias affecting radiomic feature reproducibility. Fifth, the sample size of the present study is relatively modest, with approximately 4 events per candidate predictor variable, which is below the commonly recommended threshold of 10 EPV. Although bootstrap internal validation partially mitigates overfitting concerns, the model’s performance estimates may still be optimistic. Sixth, 62 of the 359 initially screened patients (17.3%) were excluded due to loss to follow-up, which may introduce selection bias. If patients lost to follow-up differed systematically from those retained in the analysis — for example, patients with the most severe outcomes who died without follow-up documentation or those with excellent recovery who did not return for evaluation — the observed model performance could be biased in either direction. We were unable to characterize the outcome status of these excluded patients, and this represents an additional source of uncertainty.

### 4.7 Clinical implications and future directions

Subsequent research should prioritize establishing a multicenter prospective cohort covering multiple regional hospitals to collect standardized clinical and imaging data, thereby enabling rigorous external validation to assess the generalizability of the current RF model (30). The incorporation of multimodal CT/MRI imaging indicators and comprehensive laboratory biochemical parameters may expand the feature pool and potentially improve predictive performance. Automatic AI-based hematoma segmentation could replace manual delineation to reduce inter-rater variability and enhance radiomic feature reproducibility (31). Importantly, whether risk stratification guided by this radiomic model — once externally validated — translates into improved patient outcomes has not been demonstrated and warrants evaluation through prospective interventional studies. Such trials would be needed to determine whether model-informed clinical decision-making can reduce the incidence of severe long-term disability among conservatively managed BG-ICH patients (32).

## 5 Conclusion

This study constructed an interpretable CT radiomic random forest prediction model for poor 3-month functional outcome (mRS ≥ 4) in conservatively managed spontaneous basal ganglia intracerebral hemorrhage patients. Wavelet-transformed multi-scale CT texture signatures screened via LASSO regularization effectively reflect intralesional hematoma pathological heterogeneity and serve as core prognostic biomarkers. Comprehensive validation via ROC, calibration curve, DCA and KS test confirmed the RF model possesses favorable discrimination, calibration and clinical net benefit. SHAP multi-dimensional visualization clarified global feature importance and individualized risk contribution patterns, while exploratory DAG analysis and counterfactual analysis mapped hypothesized pathways linking clinical and radiomic predictors to long-term functional prognosis, generating directions for future mechanistic research. This non-invasive prediction tool, if validated in external cohorts, may support early individualized risk stratification for conservatively treated BG-ICH patients, assisting clinicians in optimizing monitoring and targeted intervention strategies for high-risk individuals. Multicenter prospective external validation and multimodal imaging optimization are required to further verify the clinical generalization of the proposed model.

## Data Availability

The original contributions presented in the study are included in the article/supplementary material, further inquiries can be directed to the corresponding author.
